# In Vivo Gastric Expression of FTO and MC4R in Sleeve Gastrectomy Patients: Diagnostic Utility Without Predictive Value for Weight Loss

**DOI:** 10.1007/s11695-025-08399-y

**Published:** 2025-12-09

**Authors:** Mohamed Hany, Mona K. ElDeeb, Ehab Elmongui, Anwar Ashraf Abouelnasr, Noha A. El-Banna, Sahar M. omer, Sara A. Shaker, Rasha A. ElTahan

**Affiliations:** 1 Department of Surgery, Alexandria Medical Research Institute, Alexandria University, Alexandria, Egypt; 2 Madina’s Bariatric Center, Madina Women’s Hospital, Alexandria, Egypt; 3 Jouf University, Sakakah, Saudi Arabia; 4 Chemical Pathology Department, Alexandria Medical Research Institute, Alexandria University, Alexandria, Egypt; 5 Independent Biostatistical Consultant, Department of Biomedical Informatics and Medical Statistics, Alexandria Medical Research Institute, Alexandria University, Alexandria, Egypt; 6 Biochemistry Department, Alexandria Medical Research Institute, Alexandria University, Alexandria, Egypt

**Keywords:** FTO, MC4R, Metabolic and bariatric surgery, Gastric gene expression, Obesity, Weight loss

## Abstract

**Background:**

The fat mass and obesity-associated (FTO) and melanocortin-4 receptor (MC4R) genes have been implicated in the pathophysiology of obesity. However, their regulatory behavior in human gastric tissue and association with postoperative weight loss following metabolic and bariatric surgery (MBS) remain unclear.

**Methods:**

In this prospective case–control study, gastric tissue from 50 patients with obesity undergoing laparoscopic sleeve gastrectomy and 48 non-obese controls was analyzed for FTO and MC4R mRNA expression using quantitative PCR. Adjusted Inverse propensity score weighting (IPSW-adjusted) and age-/sex-adjusted linear regression were applied. Receiver operating characteristic (ROC) curves were used to evaluate discriminatory thresholds. Correlation with 12-month percent total weight loss (%TWL) was assessed.

**Results:**

FTO expression was significantly upregulated (mean fold-change: 4.68, *p* < 0.001) and MC4R downregulated (mean fold-change: − 0.91, *p* < 0.001) in patients with obesity. ROC analysis identified thresholds of > 1.515 for FTO (AUC = 1.00) and < 0.525 for MC4R (AUC = 1.00), both with high sensitivity and specificity. No significant correlation was observed between gene expression and %TWL at 12-month follow-up.

**Conclusion:**

Gastric expression of FTO and MC4R accurately discriminates between individuals with and without obesity but does not predict postoperative weight loss outcomes after sleeve gastrectomy. These findings indicate diagnostic potential, whereas prognostic value remains unsubstantial.

**Supplementary Information:**

The online version contains supplementary material available at 10.1007/s11695-025-08399-y.

## Key Points

Gastric expression of FTO and MC4R was significantly different between patients with and without obesity.

FTO > 1.515-fold and MC4R < 0.525-fold thresholds effectively discriminated obesity status (AUC 1.00 for both).

No significant correlation was found between preoperative gene expression and %TWL at 12 months after sleeve gastrectomy.

FTO and MC4R may serve as diagnostic biomarkers for obesity classification, but not predictors for weight loss response.

Further studies with larger and sex-stratified cohorts are needed to validate mechanistic and prognostic roles.

**Supplementary Information:**

The online version contains supplementary material available at 10.1007/s11695-025-08399-y.

## Introduction

Obesity is a chronic, relapsing disease of energy dysregulation, resulting from sustained caloric intake exceeding individual metabolic requirements [1]. It is a major public health burden, contributing to elevated risks of type 2 diabetes, cardiovascular disease, and other obesity-associated conditions [2]. Since 1975, the global prevalence of obesity has nearly tripled, with marked increases among adults aged 18 years and older [3, 4].

Although environmental and lifestyle factors are central to obesity pathogenesis, susceptibility varies substantially across individuals. This heterogeneity reflects a complex interplay of genetic, epigenetic, and metagenomic influences [5]. From a genomic perspective, obesity can be classified into monogenic, syndromic, or polygenic forms, with the latter constituting the majority of cases [6].

Among polygenic contributors, the fat mass and obesity-associated (FTO) gene has emerged as one of the most consistently associated loci. FTO is ubiquitously expressed, including in the hypothalamic arcuate nucleus, a key integrator of energy balance and satiety signaling [7]. FTO expression has been demonstrated in the central nervous system, ventricular myocardium, pancreatic β-cells, kidney, and salivary glands in mice, pigs and human embryos [8].

Experimental studies in murine models have shown that FTO expression is sensitive to essential amino acid availability and energy status: knockdown models exhibit reduced adipose tissue ATP and are protected from diet-induced obesity, while FTO overexpression drives hyperphagia and adiposity despite normal energy expenditure [9–12]. In humans, risk alleles such as *rs9939609*, *rs3751812*, *rs9922708*, and *rs1421085* have been linked to increased energy intake and higher body mass index (BMI) [9, 13]. However, neither polymorphisms nor epigenetic markers alone have fully elucidated the gene’s mechanistic role in fat accumulation and growth regulation [13]. Moreover, FTO polymorphisms have also been linked with certain adipokines and gastrointestinal peptides [14].

Similarly, the melanocortin-4 receptor (MC4R) gene is a key regulator of energy homeostasis, primarily acting through its expression in hypothalamic neurons. As a G protein–coupled receptor, it mediates anorexigenic signals in the melanocortin system, influencing energy expenditure and appetite [15, 16].

While predominantly expressed in the hypothalamus, MC4R has also been identified in various tissues of male and female mice, including the brain, kidney, and liver, supported by qRT-PCR and immunofluorescence analyses. Additionally, studies on human neuroblastoma cell lines indicate that the SNP rs17066842 affects transcriptional activity, highlighting the complexity of MC4R’s role in energy regulation [17].

MC4R expression has also been documented in multiple peripheral tissues, including the gastrointestinal tract, as well as during fetal development in mice [15, 16, 18]. MC4R is number of five melanocortin receptors (MCRs). These MCRs have taken their order from the sequence of their cloning. MC4R was named as the neural MCRs due to its main CNS expression site. MC4R is mainly a part of energy expenditure and hunger regulation [16, 19]. Various genetic studies have been conducted on MC4R, including the study of its methylation and expression in response to a high-fat diet in an experimental mouse model, where inconsistent results were found [20].

Genetic polymorphisms in the canine melanocortin-4 receptor (MC4R) gene have been thoroughly investigated, revealing a total of 19 polymorphisms, including the notable variants p.Val103Ile and p.Ile251Leu, which confer a protective effect against obesity. Conversely, the SNP rs17782313 (C/T) has been implicated in polygenic obesity [21].

Furthermore, multiple studies have indicated that over 150 mutations in the MC4R gene are associated with monogenic forms of obesity, with the expression of MC4R mRNA detected as early as fetal development [16, 22]. In human studies, DNA methylation of the MC4R gene has been correlated with the emergence of metabolic syndrome in pediatric populations [23].

The gene expression profiles indicated a notable increase in gastric acid secretion within both obese mouse models, likely mediated by the activation of the gastrin signaling pathway. The gastrointestinal tract not only plays a pivotal role in digestive processes but also influences overall metabolic regulation through an intricate interplay among the enteric nervous system, enteroendocrine hormones, and peripheral tissue metabolism [24]. Genome-wide association studies have indicated that the FTO and MC4R genes may play a role in local gastric functions, including hormone secretion and gastric motility, potentially impacting metabolic processes within the stomach [8, 25]. However, there appears to be no direct correlation with overall body fat or systemic metabolic status. The relationship between gastric gene expression and systemic metabolic outcomes remains unexplored, presenting a potential avenue for personalized treatment strategies that target these specific genes [8, 25].

The experimental approach of evaluating obesity-related gene expression in peripheral tissues has been validated in multiple organs affected by systemic metabolic stress. Studies in adipose tissue, skeletal muscle, liver, and vascular endothelium have consistently demonstrated that obesity-associated endocrine and inflammatory signals can modulate local transcriptional activity [8, 15, 16, 25]. Extending this framework to the gastric wall provides a novel perspective on how local metabolic reprogramming may mirror systemic obesity, linking nutrient sensing and hormonal output with energy homeostasis and potential tissue-specific risk.

It is well-established that signaling pathways between the gastrointestinal tract, nutrient sensing, and the central nervous system (CNS) are crucial in regulating appetite and energy balance. Upon food detection, sensory and hormonal signals from the stomach are transmitted to hypothalamic centers, where neurotransmitters and hormones interact with MC4R to suppress hunger [26]. Moreover, variants in the MC4R gene have been linked to diminished sensitivity to ghrelin, a key appetite-regulating hormone [27].

Numerous studies have examined the associations between single-nucleotide variants (SNVs), genetic risk scores, and weight loss; however, findings remain inconsistent across the literature [28, 29]. Importantly, several common variants in both genes act as expression quantitative trait loci (eQTLs). For FTO, intronic SNPs such as rs9939609 modulate expression of FTO and downstream regulators of adipogenesis, including IRX3 and IRX5 [30–33]. For MC4R, variants such as rs17782313 have been linked to altered transcriptional activity and reduced melanocortin signaling in appetite-regulating pathways [27, 34–36].

To date, no studies have examined human gastric gene expression of FTO and MC4R as predictors of obesity status or weight loss following metabolic and bariatric surgery (MBS). This study aimed to evaluate gastric FTO and MC4R gene expression in patients with and without obesity, and to assess their association with the percentage of total weight loss (%TWL) following MBS. This approach may offer insight into the utility of local tissue biomarkers for stratifying patients and tailoring surgical interventions in obesity management.

We hypothesized that gastric expression of FTO and MC4R would differ between individuals with and without obesity, reflecting obesity-associated alterations in peripheral metabolic regulation. This study, therefore, aimed to (1) quantify gastric FTO and MC4R mRNA expression in patients with obesity versus non-obese controls, and (2) explore whether these expression profiles relate to postoperative weight-loss response following sleeve gastrectomy. The primary objective focused on discriminatory (diagnostic) potential, whereas postoperative correlations were evaluated as an exploratory outcome rather than a predictive hypothesis. These analyses generate mechanistic insight into tissue-specific obesity biology but do not constitute a validated clinical prediction model.

## Methods

### Study Design and Participants

This prospective, single-center case–control study was conducted at the Alexandria Medical Research Institute, Alexandria University, Alexandria, Egypt. A total of 98 participants were enrolled: 50 patients with obesity scheduled for primary metabolic and bariatric surgery (MBS), and 48 non-obese controls undergoing elective diagnostic upper gastrointestinal endoscopy.

Obesity status was classified using BMI alone, consistent with the World Health Organization (WHO) definition and the 2022 American Society of Metabolic and Bariatric Surgery (ASMBS) and the International Federation for the Surgery and Other Therapies for Obesity (IFSO) guidelines, whereby a BMI ≥ 30 kg/m² indicates clinical obesity [37, 38]. Individuals with a BMI of < 30 kg/m² were not categorized as having obesity and were taken as controls.

Participants were excluded if they had any gastric pathology, including *Helicobacter pylori* infection, malignancy, patients with endocrine or psychiatric disease, or were receiving medications that could influence the metabolic function, including corticosteroids, thiazolidinediones, ACE inhibitors, ARBs, clonidine, or fenofibrate.

All patients in the MBS group underwent laparoscopic sleeve gastrectomy (SG) and were followed for total weight loss (%TWL) at 12 months. None had undergone prior MBS or gastric surgery.

The study was approved by the institutional ethics committee of the Medical Research Institute (approval code: E/C.S/N.R12/2024), and written informed consent was obtained from all participants.

### Anthropometric and Clinical Assessments

Body weight and height were measured using standardized protocols, and BMI was calculated as weight in kilograms divided by height in meters squared (kg/m²). WC and WHR were measured using non-elastic tape according to standard anthropometric procedures. Fasting venous blood samples were collected intraoperatively and analyzed for lipid profile, fasting blood glucose (FBG), insulin, and homeostatic model assessment for insulin resistance (HOMA-IR).

### Pre-operative Esophagogastroduodenoscopy

All participants underwent esophagogastroduodenoscopy (EGD) before tissue sampling. In the SG group, EGD was performed routinely during the preoperative evaluation to confirm surgical eligibility and exclude pathological findings; only patients with negative endoscopic findings were included. In the non-obese group, patients were selected from individuals scheduled to undergo routine EGD for other clinical indications, primarily abdominal pain or gastroesophageal reflux-like symptoms, as part of standard hospital care. Only individuals with macroscopically negative EGD findings were enrolled in the study.

A standardized biopsy protocol was applied to all participants, involving three passes to obtain six antral mucosal specimens. All biopsies were fixed in 10% buffered formalin, embedded in paraffin, and sent for RNA extraction and PCR evaluation.

### Surgical Procedure

All SG procedures were performed by a single experienced surgical team using a standardized laparoscopic approach. Pneumoperitoneum was established using optical trocar entry, followed by placement of five ports: three 12-mm ports (camera and bilateral working ports) and two 5-mm ports (for liver retraction and assistance).

The greater curvature of the stomach was mobilized beginning approximately 3–5 cm from the pylorus and extending to the angle of His. The greater omentum was dissected using the EnSeal device (Ethicon Endo-Surgery, Cincinnati, OH, USA), and any posterior gastric adhesions were released. Belsey’s pad of fat was excised when present to facilitate exposure.

Gastric calibration was achieved using a 36-Fr bougie placed along the lesser curvature. Gastric transection was performed using the Echelon Flex Endopath 60-mm linear stapler (Ethicon Endo-Surgery, Cincinnati, OH, USA), employing green, gold, and blue cartridges according to tissue thickness.

The staple line was fully invaginated with a running seromuscular suture using unidirectional absorbable 3/0 V-Loc™ 180 sutures (Covidien, Mansfield, MA, USA).

### Gastric Tissue Collection and RNA Extraction

Gastric mucosal biopsies were obtained from the antral mucosa during EGD and were immediately processed for RNA extraction without chemical fixation to preserve RNA integrity. To minimize circadian and acute nutritional influences on gene expression, all samples from both groups were collected endoscopically in the morning after a standardized overnight fast, ensuring identical tissue-sampling conditions for both groups. Total RNA was extracted using the RNeasy Mini Kit (Qiagen, Hilden, Germany), and RNA purity was verified by measuring the A260/A280 ratio using a Nanodrop ND-1000 spectrophotometer.

### cDNA Synthesis and Quantitative PCR

Complementary DNA (cDNA) was synthesized using the QuantiTect Reverse Transcription Kit (Qiagen, Cat. No. 205311), following the manufacturer’s instructions. Quantitative real-time PCR (qPCR) was performed using the Rotor-Gene SYBR Green PCR Kit (Qiagen, USA). Pre-designed QuantiNova LNA PCR assays were used to quantify FTO (GeneGlobe ID: SBH0350496) and MC4R (GeneGlobe ID: SBH0618803) expression, with GAPDH (SBH0555536) as the reference gene. Amplification was initiated with denaturation at 95 °C for 10 min, followed by 40 cycles at 95 °C for 5 s, 55 °C for 15 s, and 60 °C for 15 s. Gene expression levels were normalized to GAPDH and calculated using the 2^−ΔΔCt method [39, 40].

### Sample Size Calculation

The sample size was determined a priori based on the primary outcome, which was the difference in gastric FTO and MC4R expression between patients with obesity and non-obese controls. Using GPower version 3.1.9.7, we calculated that detecting a large effect size (Cohen’s d = 0.80) with a two-tailed independent t-test, α = 0.05, and 80% power would require 26 participants per group. To strengthen the study and allow for adjustments using inverse propensity score weighting, we recruited 50 cases and 48 controls (total *N* = 98).

### Statistical Analysis

Descriptive statistics were used to summarize the demographic and clinical characteristics of the participants. Continuous variables were presented as mean ± standard error (SE) and compared between obese cases and non-obese controls using linear regression models. Categorical variables were expressed as counts and percentages and compared using weighted chi-square tests.

To minimize confounding, inverse propensity score weighting (IPSW) was applied to balance age and sex between cases and controls while preserving differences in anthropometric variables. This was facilitated by the Toolkit for Weighting and Analysis of Nonequivalent Groups (TWANG) package (2). We implemented binomial inverse propensity-score weighting utilizing a gradient boosting machine (GBM) model in an Average Treatment effect on the Treated (ATT) framework. We determined the optimal number of GBM trees by assessing the plot of average absolute standardized mean difference (aSMD) across covariates against the number of iterations (S1), finding that the balance was optimally achieved at 240 trees. The enhancement in balance across the groups was confirmed through plots of absolute Standardized Mean Differences (aSMD) and p values (S2 and S3), with aSMD values < 0.1 indicating optimal balance.

For metabolic profile and gene expression analyses, mean differences (MD) and 95% confidence intervals (CI) were computed for unadjusted comparisons using standard linear regression models. After the IPSW application, adjusted mean differences (AMD) with 95% CI were estimated using weighted regression models to account for the stabilized weights. Further adjustments for age and sex were applied following IPSW in a double-adjusted analysis to ensure robustness. All 98 participants contributed fully to the weighted analyses.

To further explore the relationship between gene expression and metabolic markers, additional weighted linear regression models were constructed with FTO and MC4R expression levels as independent variables and key metabolic parameters as dependent variables, adjusting for case status. The same models were repeated without applying weights, and results were presented in the supplementary material for comparison.

To examine associations between FTO and MC4R gene expression levels and BMI, Pearson correlation coefficients (r) with corresponding p-values were computed separately for cases and controls. The discriminatory ability of FTO and MC4R expression levels in distinguishing between obese and non-obese individuals was evaluated using receiver operating characteristic (ROC) curve analysis. The optimal expression thresholds were determined based on the highest Youden’s index, and the corresponding area under the curve (AUC), sensitivity, and specificity were reported with 95% CIs. Statistical analyses were performed using R software (version 4.4.2) and MedCalc version 12.4.0.0, and a p-value < 0.05 was considered statistically significant.

## Results

### Baseline Characteristics and Propensity Score Adjustment

A total of 98 participants were included in the analysis, comprising 50 patients with obesity undergoing metabolic and bariatric surgery (MBS) and 48 non-obese controls undergoing diagnostic endoscopy. Before adjustment, the obesity group was significantly younger than controls (31.4 ± 1.5 vs. 50.5 ± 2.2 years; *p* < 0.001; aSMD = 0.87). Following inverse propensity score weighting (IPSW), the age difference was attenuated and no longer statistically significant (31.4 ± 1.5 vs. 34.7 ± 2.4 years; *p* = 0.255; aSMD = 0.27), though the aSMD remained above the 0.1 threshold for optimal balance.

Sex distribution was comparable between groups before and after weighting (*p* = 0.281 unadjusted; *p* = 0.994 IPSW), and aSMD was reduced to 0.00, indicating excellent balance. As expected, body weight and BMI remained significantly higher in the MBS group (118.3 ± 3.2 kg and 43.1 ± 0.9 kg/m²) compared to controls (66.8 ± 2.4 kg and 25.0 ± 0.6 kg/m²), with p-values < 0.001 and aSMD > 2.8 (Table 1).

**Table 1:** Demographic data of the participants

Variable	Unadjusted analysis				IPSW adjusted analysis			
	Patients with Obesity *n* = 50	Patients without obesity (Controls) *n* = 48	*P*	aSMD	Patients with Obesity *n* = 50	Patients without obesity (Controls) *n* = 33	*p*	aSMD
Age (years)	31.4 ± 1.5	40.5 ± 2.2	**< 0.001**	0.87	31.4 ± 1.5	34.7 ± 2.4	0.255	0.27
Sex								
Male, n (%)	9 (18)	13 (27.1)	0.281	0.24	9 (18)	6 (17.9)	0.994	0.00
Female, n (%)	41 (82)	35 (72.9)			41 (82)	27 (82.1)		
Anthropometrics								
Height (cm)	165.4 ± 1.3	163.0 ± 0.9	0.127	0.31	165.4 ± 1.3	163.0 ± 1.4	0.214	0.29
Weight (Kg)	118.3 ± 3.2	68.4 ± 1.5	**< 0.001**	2.86	118.3 ± 3.2	66.8 ± 2.4	< 0.001*	2.89
BMI (Kg/m^2^)	43.1 ± 0.9	25.7 ± 0.4	**< 0.001**	3.46	43.1 ± 0.9	25.0 ± 0.6	< 0.001*	3.59

Data are presented as mean ± SD, or n (%), as appropriate. BMI: body mass index (kg/m²); Comparisons were adjusted using inverse propensity score weighting (IPSW). *p < 0.05. Weighted values represent effective sample estimates based on stabilized weights.

### Metabolic Profile and Gene Expression: IPSW and Double-Adjusted Models

After IPSW adjustment, fasting blood glucose (FBG) was significantly higher in the MBS group (AMD = 10.23 mg/dL; 95% CI: 5.07 to 15.39; *p* < 0.001), and this association persisted after double adjustment for age and sex (AMD = 10.66 mg/dL; 95% CI: 5.09 to 16.23; *p* < 0.001). The cholesterol-to-HDL ratio was also significantly higher after double adjustment (AMD = 0.61; 95% CI: 0.02 to 1.20; *p* = 0.043). Other lipid indices and insulin resistance markers, including HOMA-IR and LDL/HDL, showed no significant differences between groups (Table 2).

**Table 2:** Differences between the patients with obesity and non-obese controls in terms of their metabolic profile, appetite, and genetic expression of FTO and MC4R in the IPSW adjusted and the double adjusted analyses

Variable	IPSW Adjusted		Double-adjusted	
	AMD (95% CI)	*p*	AMD (95% CI)	*p*
**Metabolic profile:**				
Total lipid (mg/dL)	−7.49 (−53.65, 38.68)	0.748	3.87 (−40.05, 47.79)	0.861
Triglycerides (mg/dL)	−3.70 (−22.52, 15.12)	0.697	−0.15 (−17.42, 17.12)	0.986
Cholesterol (mg/dL)	3.19 (−15.65, 22.03)	0.738	7.04 (−11.04, 25.12)	0.441
LDL (mg/dL)	0.79 (−15.51, 17.08)	0.924	3.86 (−11.13, 18.84)	0.610
HDL (mg/dL)	4.26 (−0.25, 8.76)	0.064	4.30 (−0.53, 9.13)	0.080
Cholesterol/HDL	0.57 (−0.02, 1.16)	0.060	0.61 (0.02, 1.20)	0.043*
LDL/HDL	−0.29 (−0.69, 0.12)	0.165	−0.22 (−0.57, 0.14)	0.230
Insulin (micro-IU/mL)	−1.62 (−4.78, 1.54)	0.312	−2.01 (−4.90, 0.88)	0.171
HOMA-IR	−0.15 (−0.90, 0.60)	0.696	−0.23 (−0.93, 0.47)	0.514
FBG (mg/dL)	10.23 (5.07, 15.39)	< 0.001*	10.66 (5.09, 16.23)	< 0.001*
**Genetic expression:**				
MC4R (fold change)	−0.89 (−1.07, −0.71)	< 0.001*	−0.91 (−1.07, −0.74)	< 0.001*
FTO (fold change)	4.75 (3.90, 5.60)	< 0.001*	4.68 (3.88, 5.47)	< 0.001*

Cell values represent adjusted mean differences (AMD) with 95% confidence intervals (CI) unless otherwise specified. IPSW: inverse propensity score weighting. *p < 0.05. Double-adjusted values refer to additional adjustment for age and sex following IPSW. HDL: high-density lipoprotein cholesterol (mg/dL); LDL: low-density lipoprotein cholesterol (mg/dL); Cholesterol/HDL and LDL/HDL denote lipid ratios; FBG: fasting blood glucose (mg/dL); HOMA-IR: homeostatic model assessment for insulin resistance. FTO: fat mass and obesity-associated gene; MC4R: melanocortin-4 receptor gene. Fold-change values calculated using the 2⁻ΔΔCt method and normalized to GAPDH

FTO expression was significantly upregulated in the MBS group, with an adjusted mean fold-change difference of 4.75 (95% CI: 3.90 to 5.60; *p* < 0.001) in the IPSW model and 4.68 (95% CI: 3.88 to 5.47; *p* < 0.001) after double adjustment. MC4R expression was significantly downregulated in both models (IPSW AMD = − 0.89; 95% CI: − 1.07 to − 0.71; *p* < 0.001; double-adjusted AMD = − 0.91; 95% CI: − 1.07 to − 0.74; *p* < 0.001) (Table 2).

### Discrimination between Obesity and Non-Obesity by Gene Expression

**Fig. 1:**
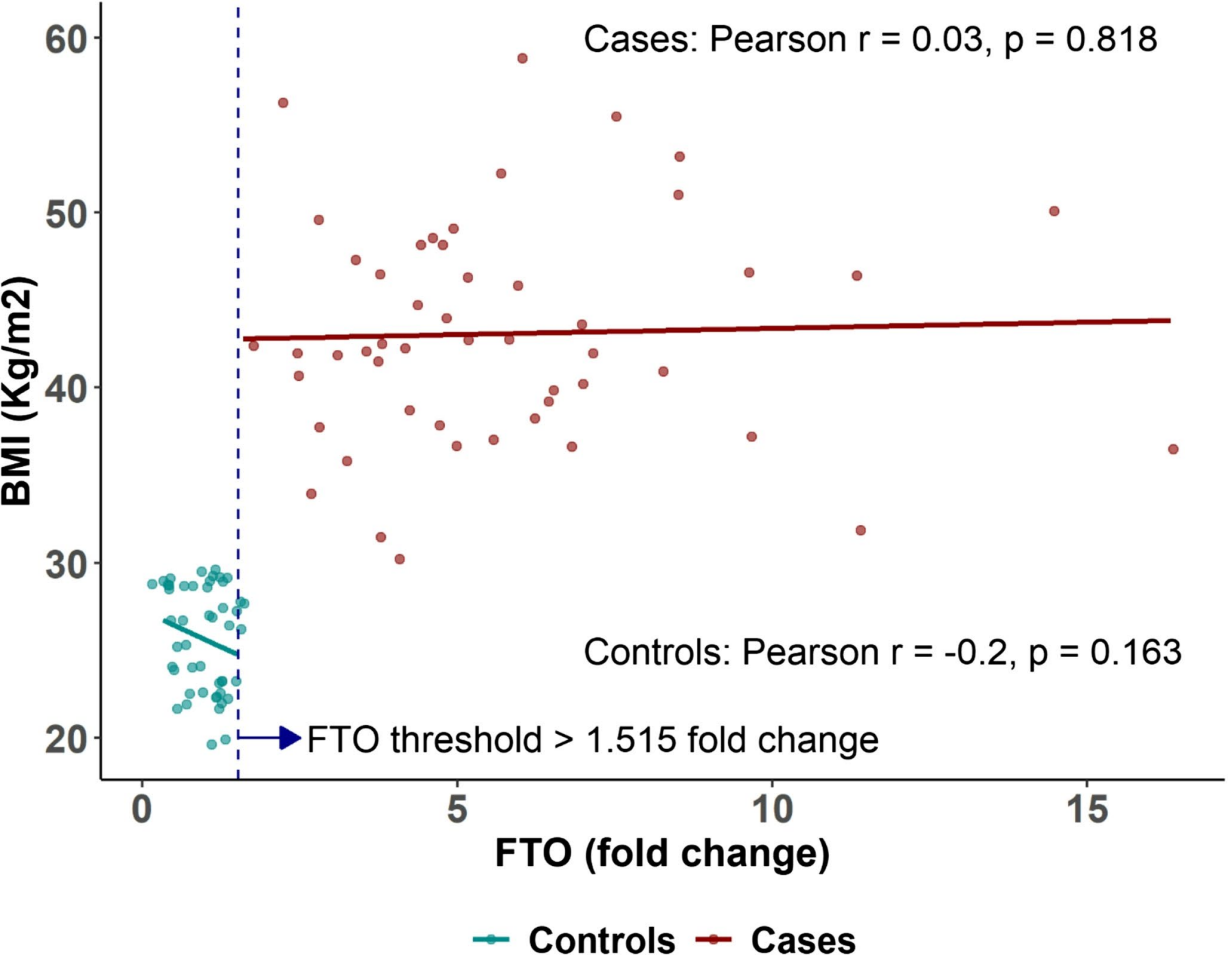
Scatter plot showing the relationship between FTO gene expression (fold change) and BMI in patients with obesity (red) and non-obese controls (cyan). Pearson correlation coefficients and p-values are displayed for each group. The FTO threshold > 1.515-fold change, identified using ROC curve analysis, is indicated by the vertical dashed line. This threshold demonstrated perfect discrimination between cases and controls, as shown in Supplementary Figure S1

**Fig. 2:**
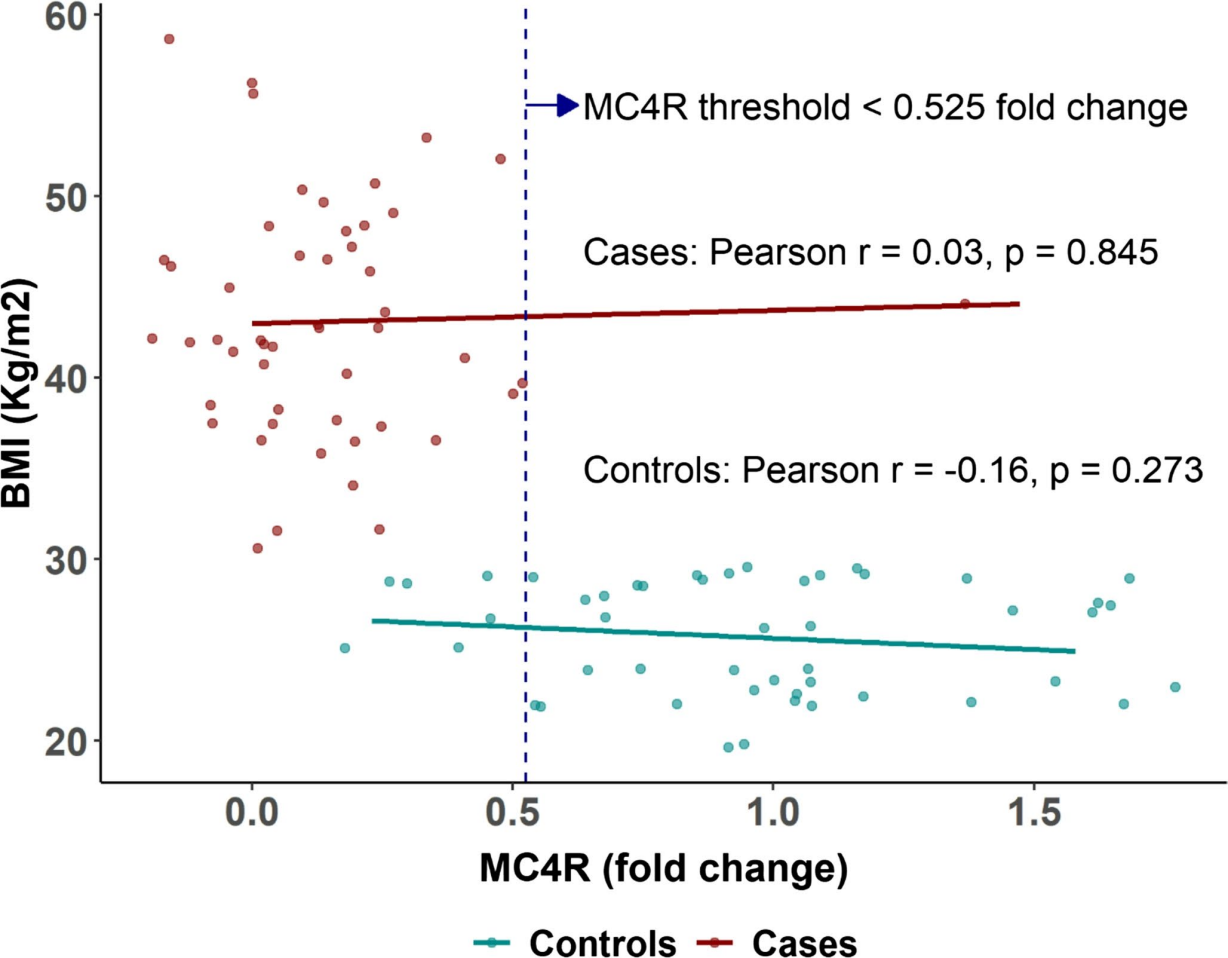
Scatter plot illustrating the relationship between MC4R gene expression (fold change) and BMI in patients with obesity (red) and non-obese controls (cyan). Pearson correlation coefficients and p-values are shown for each group. The MC4R threshold < 0.525-fold change, identified using ROC curve analysis, is indicated by the vertical dashed line. This threshold showed strong discriminatory power in differentiating obesity status, as demonstrated in Supplementary Figure S2

Clear separation in FTO and MC4R expression levels was observed between the two groups (Figs. 1 and 2). No significant correlation was detected between FTO expression and BMI among patients with obesity (*r* = 0.03, *p* = 0.818) or controls (*r* = − 0.20, *p* = 0.168). A fold-change threshold of > 1.515 for FTO yielded an AUC of 1.00 (95% CI: 1.00 to 1.00), with both sensitivity and specificity reaching 100% (95% CI: 92.9 to 100%) (Supplementary Figure S1).

MC4R expression was also not correlated with BMI (*r* = 0.03, *p* = 0.845 in cases; *r* = − 0.06, *p* = 0.671 in controls). A threshold of < 0.525-fold change provided an AUC of 0.97 (95% CI: 0.93 to 1.00), sensitivity of 98% (95% CI: 89.4 to 99.9%), and specificity of 88% (95% CI: 75.7 to 95.5%) (Supplementary Figure S2).

Within the control group of non-obese participants (BMI < 30), no significant differences were observed in FTO or MC4R expression between those with normal BMI (< 25) and those classified as overweight (25–29.9). Mean FTO expression was 1.01 ± 0.34 in the normal group and 0.90 ± 0.39 in the overweight group (*p* = 0.301). MC4R expression was 1.04 ± 0.32 in the normal group and 0.89 ± 0.43 in the overweight group (*p* = 0.161).

### Association between Gene Expression and Postoperative Weight Loss

**Fig. 3:**
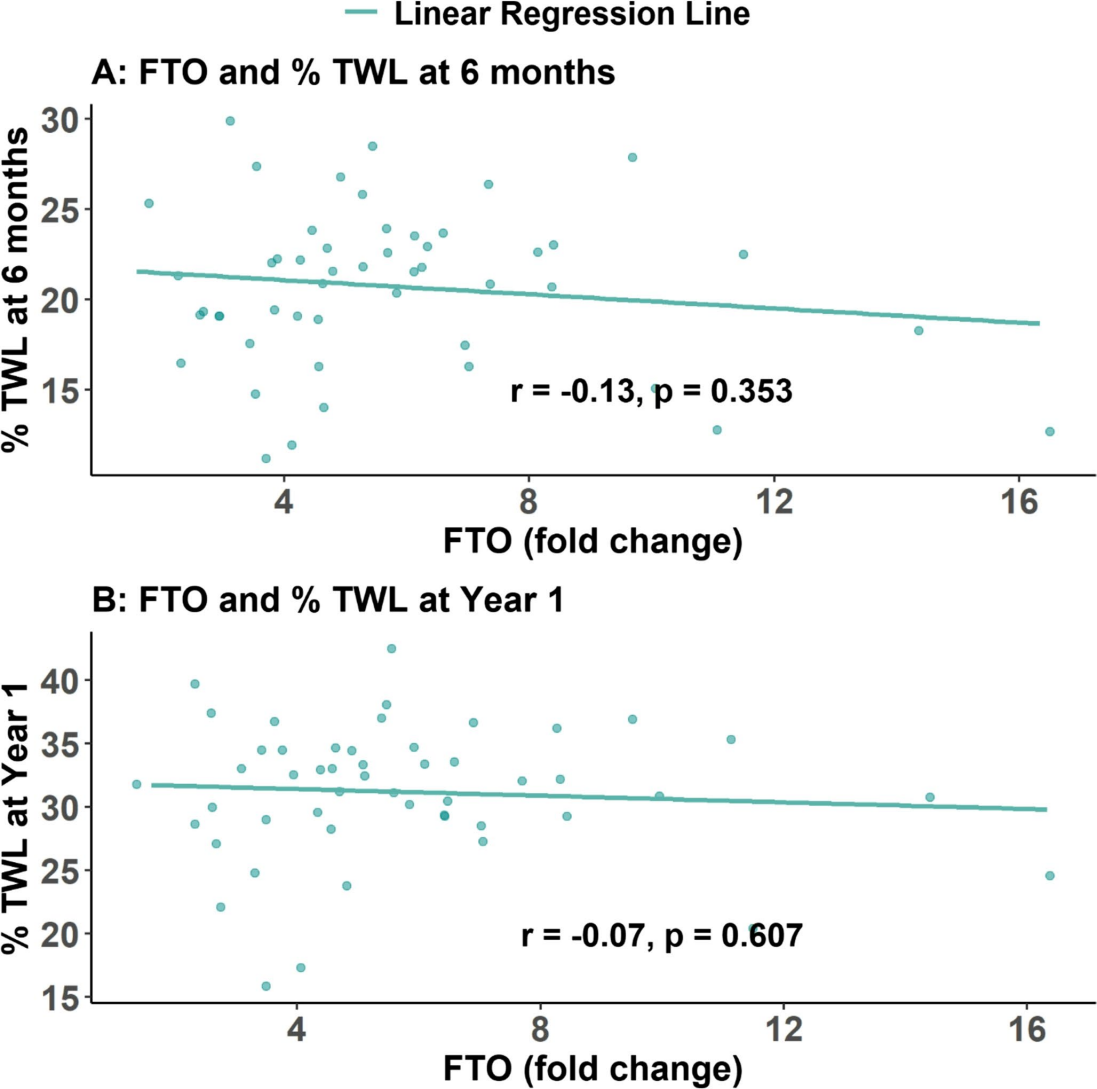
Scatter plots showing the association between FTO gene expression (fold change) and percentage of total weight loss (%TWL) among patients with obesity who underwent MBS. A lack of correlation was observed at 6 months (*r* = − 0.13, *p* = 0.353) or at 1 year (*r* = − 0.07, *p* = 0.607). A linear regression line is overlaid to illustrate the direction of association at both time points

**Fig. 4:**
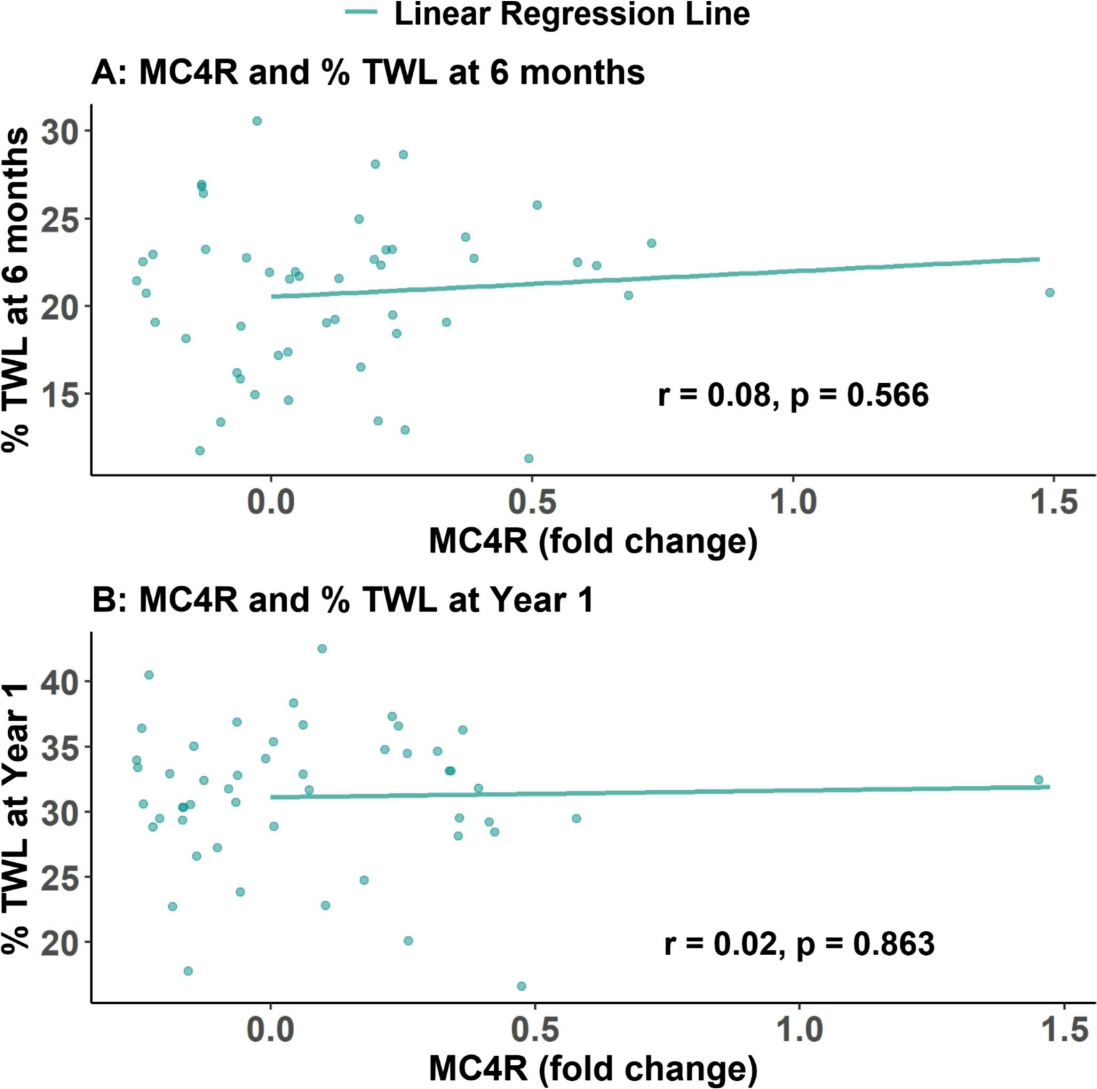
Scatter plots show the association between MC4R gene expression (fold change) and percentage of total weight loss (%TWL) among patients with obesity who underwent MBS. No significant correlation was observed at 6 months (*r* = 0.08, *p* = 0.566) or at 1 year (*r* = 0.02, *p* = 0.863). Linear regression lines are overlaid to illustrate the direction of association at both time points

A lack of correlation was observed between FTO expression and %TWL at 6 months (*r* = − 0.13, *p* = 0.353) or at 1 year (*r* = − 0.07, *p* = 0.607) (Fig. 3). Similarly, MC4R expression was not associated with %TWL at 6 months (*r* = 0.08, *p* = 0.566) or at 1 year (*r* = 0.02, *p* = 0.863) (Fig. 4). These results confirm that extending follow-up to 1 year does not alter the absence of correlation between baseline gene expression and postoperative weight-loss outcomes.

## Discussion

Genetic factors play a critical role in the pathogenesis of obesity, functioning independently or in interaction with various other elements, such as additional genetic variants, environmental factors, and hormonal regulators [5, 6]. Familial studies first identified a positive correlation between genetic predisposition and obesity, revealing a higher prevalence in monozygotic twins compared to dizygotic twins [41, 42].

Advancements in genome-wide association studies (GWAS) and next-generation sequencing (NGS) have refined our understanding of genetic predictors of obesity [41, 43]. Investigating the genetic underpinnings linked to weight loss following MBS may pave the way for the development of novel weight-loss pharmacotherapies [44].

Among various genes, the FTO gene has emerged as a key player in obesity susceptibility, exhibiting the strongest association with the obesity phenotype [45, 46]. The FTO risk variant has been associated with IRX3 expression and adipocyte hypertrophy, particularly in lean children, suggesting a potential protective mechanism for body weight regulation [33].

Genome-wide association studies further suggest that FTO and MC4R may influence local gastric functions, such as hormone secretion, motility, and nutrient sensing, that participate in metabolic regulation without necessarily mirroring overall adiposity [8, 25]. This raises the possibility that gastric FTO and MC4R expression reflects tissue-specific metabolic adaptations rather than purely systemic effects.

Beyond obesity, increasing evidence links FTO overexpression to gastrointestinal malignancies. Acting as an N⁶-methyladenosine (m⁶A) demethylase, FTO removes m⁶A marks from oncogenic transcripts such as MYC, enhancing their stability and translation. In gastric and colorectal cancer, this mechanism promotes tumor cell proliferation, migration, and invasion [47]. Although all participants with malignancy were excluded from our cohort and cancer pathways were beyond our study scope, these data suggest that gastric FTO expression could represent a molecular bridge between chronic metabolic stress and carcinogenic susceptibility, a hypothesis meriting future dedicated investigation.

Additionally, FTO has been implicated in several metabolic pathways associated with obesity, including polycystic ovary syndrome (PCOS), type 2 diabetes mellitus (T2DM), and cancer [48]. Environmental modifications and certain substances can influence FTO expression, indicating that targeting FTO pathways may offer therapeutic avenues for FTO-related obesity [49].

Research has explored the epigenetics of FTO, SNPs, and gene expression in both human and animal models of obesity [8, 49]. Notably, the FTO genotype (AA) for rs9939609 was established as a positive predictor of weight gain in the Japanese population, correlating with a heightened risk of weight regain during a five-year follow-up period post-intervention [50].

The MC4R, a G protein-coupled receptor, possesses 61 gene variants associated with obesity and its related metabolic and cardiovascular complications through diverse signaling pathways, positioning it as a target for personalized obesity therapies [51]. Comprehensive analysis of MC4R SNPs has revealed associations with obesity across various geographic regions and age demographics, particularly for variants rs17782313 and rs12970134 [52].

Whole-exome sequencing (WES) identified a heterozygous MC4R: c.216 C > G (p.Asn72Lys) variant linked to a case of severe early-onset monogenic obesity accompanied by metabolic complications, warranting further confirmatory studies and fostering hope for individualized treatment modalities [53]. Additionally, the rs17782313 MC4R variant has been associated with emotional eating behaviors, highlighting its potential role in targeting psychological components of obesity through agonist treatments [54].

Studies examining FTO genotypes (rs9939609, rs9930506, and rs1421085) and MC4R rs17782313 polymorphisms with total body weight loss (TBWL), post-operative weight, and post-MBS BMI have shown a significant correlation exclusively between FTO risk genotypes rs9930506 and rs1421085 and weight loss outcomes [55].

According to our knowledge, human studies assessing FTO and MC4R gastric gene expression as predictors of obesity and %TWL post-MBS remain sparse. Our findings indicate that an FTO gastric gene expression threshold > 1.515 fold change serves as the optimal cutoff with an AUC of 1.00 (95% CI: 1.00 to 1.00), achieving 100% sensitivity and specificity (95% CI: 92.9 to 100%). While a lack of correlation was observed between FTO expression and %TWL at 6 months (*r* = − 0.13, *p* = 0.353) or at 1 year (*r* = − 0.07, *p* = 0.607). Nonetheless, FTO gastric expression remains a potential target for small-molecule drugs and micronutrients aimed at modulating its expression, offering a new therapeutic approach for obesity and related metabolic disorders [8].

In terms of MC4R gastric gene expression, although no significant association with BMI was found in either cases or controls, a cutoff of < 0.525-fold change effectively distinguished between obese and non-obese individuals. Additionally, a lack of correlation was identified between MC4R gastric expression and %TWL at 6 months (*r* = 0.08, *p* = 0.566) or at 1 year (*r* = 0.02, *p* = 0.863).

Thus, while gastric expression of FTO and MC4R effectively distinguishes individuals with obesity from those without obesity, these markers did not demonstrate predictive utility for postoperative weight loss outcomes following MBS. These findings support a tissue-specific role for FTO and MC4R in the pathophysiology of obesity. Unlike adipose or hypothalamic expression, gastric expression likely reflects local adaptations to chronic nutrient exposure, altered gastric endocrine signaling, and inflammatory remodeling associated with obesity. Accordingly, our primary interpretation focuses on their diagnostic discrimination of obesity status rather than the mechanistic prediction of postoperative response. The lack of correlation with weight-loss outcomes indicates that gastric transcriptional alterations represent a biological marker of the obese state rather than a determinant of therapeutic success, reinforcing the conceptual distinction between diagnostic and prognostic applications.

### Strengths and Limitations

These findings should be interpreted considering certain limitations. The single-center design and modest sample size may limit generalizability, although the use of inverse propensity score weighting and double-adjusted models reduced confounding risk. Gene expression was assessed exclusively at the mRNA level without corresponding protein validation, and no immunohistochemistry or Western blot analyses were performed, which limits direct inference about translational effects. Additionally, quantification was based on the 2⁻ΔΔCt method, which reflects relative fold-changes rather than absolute transcript abundance; while appropriate for group comparisons, this approach may incompletely represent biological differences in expression magnitude. Although age and sex were balanced between groups and included in double-adjusted models, lifestyle factors such as smoking were not recorded and may contribute to residual variability. Supporting this possibility, nicotine exposure has been shown to modulate MC4R transcription in mammalian neural tissue [56], and disease-associated dysregulation of FTO expression has been reported in human cell studies [57]. Whether similar mechanisms operate in normal gastric mucosa remains unknown. Nevertheless, current evidence linking these factors to gene regulation derives largely from adipose tissue and animal models, while data in normal human gastric mucosa remain sparse [58–60].

Importantly, gastric expression of FTO and MC4R should be interpreted as a marker of local metabolic adaptation rather than a direct proxy for central or systemic energy-balance regulation, and future multimodal studies are needed to clarify how these local transcriptional changes integrate within broader obesity pathophysiology. Moreover, detailed obesity-related diseases and medication profiles were not systematically collected, which limits full clinical contextualization of gastric gene-expression findings and should be addressed in future studies. Furthermore, the number of male participants was relatively low, which may limit the power to detect sex-specific effects in gastric gene expression and postoperative outcomes despite statistical adjustment. Future studies should incorporate sex-stratified recruitment and analyses to evaluate potential differential responses better. Future studies should therefore incorporate standardized assessments of lifestyle behavior, comorbid disease, medication exposure, and epigenetic parameters, alongside protein-level evaluation, to validate and contextualize these molecular findings. Finally, postoperative follow-up was limited to a 12-month interval, restricting evaluation of long-term metabolic trajectories after MBS.

Strengths of the study include its prospective design, inclusion of well-matched non-obese controls, standardized gastric sampling, and rigorous statistical methodology. The use of validated TaqMan qPCR assays with blinded triplicate analysis ensured high analytical precision. To our knowledge, this is one of the first studies to evaluate in vivo gastric FTO and MC4R expression with obesity and weight loss outcomes following MBS.

### Future Directions

Future research should focus on validating these findings across larger and more diverse cohorts, extending follow-up to capture long-term outcomes, and incorporating complementary molecular layers, particularly DNA methylation, miRNA regulation, and histone modification, to elucidate the epigenetic mechanisms governing gastric FTO and MC4R expression. The observed mRNA differences may reflect m⁶A demethylase-mediated reprogramming of local metabolism by FTO or miRNA-driven suppression of MC4R, mechanisms that remain to be explored in human gastric tissue. Moreover, given the emerging association between FTO overexpression and gastrointestinal oncogenesis [47], future studies should determine whether the gastric transcriptional alterations observed in obesity also signify increased tissue vulnerability to carcinogenic transformation.

From a translational standpoint, pharmacologic modulation of these pathways remains in early development. FTO demethylase inhibitors and selective MC4R agonists have shown promise as precision-medicine approaches for obesity and metabolic disorders [61, 62], highlighting the therapeutic relevance of these genes as multi-target regulatory hubs.

## Conclusion

Gastric expression of the FTO and MC4R genes demonstrates strong discriminatory ability between individuals with and without obesity in our cohort, suggesting a potential role in gastric molecular phenotyping of obesity. However, neither marker showed predictive value for postoperative weight-loss response following metabolic and bariatric surgery. These findings position gastric FTO and MC4R expression as diagnostic rather than prognostic candidates in obesity care. Further validation in larger, longitudinal cohorts is warranted.

## Supplementary Information

Below is the link to the electronic supplementary material.

Supplementary Material 1 (DOCX 139 KB)

## Data Availability

The datasets generated and/or analyzed during the current study are available from the corresponding author on request.

## References

[1] Hall KD, Farooqi IS, Friedman JM, Klein S, Loos RJF, Mangelsdorf DJ, O’Rahilly S, Ravussin E, Redman LM, Ryan DH, et al. The energy balance model of obesity: beyond calories in, calories out. Am J Clin Nutr. 2022;115(5):1243–54. 10.1093/ajcn/nqac031. 35134825 10.1093/ajcn/nqac031 PMC9071483

[2] Piché ME, Tchernof A, Després JP. Obesity phenotypes, diabetes, and cardiovascular diseases. Circ Res. 2020;126(11):1477–500. 10.1161/circresaha.120.316101. 32437302 10.1161/CIRCRESAHA.120.316101

[3] obesity and overweight WHO world health organization. 2021, Available at: https://www.who.int/news-room/fact-sheets/detail/obesity-and-overweight. Accessed 15 Jun 2023.

[4] Safaei M, Sundararajan EA, Driss M, Boulila W, Shapi’i A. A systematic literature review on obesity: understanding the causes & consequences of obesity and reviewing various machine learning approaches used to predict obesity. Comput Biol Med. 2021;136:104754. 10.1016/j.compbiomed.2021.104754. 34426171 10.1016/j.compbiomed.2021.104754

[5] Mackenbach JD, Rutter H, Compernolle S, Glonti K, Oppert JM, Charreire H, De Bourdeaudhuij I, Brug J, Nijpels G, Lakerveld J. Obesogenic environments: a systematic review of the association between the physical environment and adult weight status, the SPOTLIGHT project. BMC Public Health. 2014;14:233. 10.1186/1471-2458-14-233. 24602291 10.1186/1471-2458-14-233 PMC4015813

[6] Thaker VV, GENETIC, AND EPIGENETIC CAUSES OF OBESITY. Adolesc Med State Art Rev. 2017;28(2):379–405. 30416642 PMC6226269

[7] Asuquo EA, Nwodo OFC, Assumpta AC, Orizu UN, Oziamara ON, Solomon OA. *FTO* gene expression in diet-induced obesity is downregulated by *solanum* fruit supplementation. Open Life Sci. 2022;17(1):641–58. 10.1515/biol-2022-0067. 35800074 10.1515/biol-2022-0067 PMC9202533

[8] Huang C, Chen W, Wang X. Studies on the fat mass and obesity-associated (*FTO*) gene and its impact on obesity-associated diseases. Genes Dis. 2023;10(6):2351–65. 10.1016/j.gendis.2022.04.014. 37554175 10.1016/j.gendis.2022.04.014 PMC10404889

[9] Harbron J, van der Merwe L, Zaahl MG, Kotze MJ, Senekal M. Fat mass and obesity-associated (FTO) gene polymorphisms are associated with physical activity, food intake, eating behaviors, psychological health, and modeled change in body mass index in overweight/obese Caucasian adults. Nutrients. 2014;6(8):3130–52. 10.3390/nu6083130. 25102252 10.3390/nu6083130 PMC4145299

[10] Speakman JR. The ‘fat mass and obesity related’ (FTO) gene: mechanisms of impact on obesity and energy balance. Curr Obes Rep. 2015;4(1):73–91. 10.1007/s13679-015-0143-1. 26627093 10.1007/s13679-015-0143-1

[11] Peng S, Xiao W, Ju D, Sun B, Hou N, Liu Q, Wang Y, Zhao H, Gao C, Zhang S, et al. Identification of Entacapone as a chemical inhibitor of FTO mediating metabolic regulation through FOXO1. Sci Transl Med. 2019;11(488). 10.1126/scitranslmed.aau7116. 10.1126/scitranslmed.aau7116 30996080

[12] Merkestein M, McTaggart JS, Lee S, Kramer HB, McMurray F, Lafond M, et al. Changes in gene expression associated with FTO overexpression in mice. PLoS One. 2014;9(5):e97162. 10.1371/journal.pone.0097162. 24842286 10.1371/journal.pone.0097162 PMC4026227

[13] Franzago M, Fraticelli F, Marchioni M, Di Nicola M, Di Sebastiano F, Liberati M, Stuppia L, Vitacolonna E. Fat mass and obesity-associated (FTO) gene epigenetic modifications in gestational diabetes: new insights and possible pathophysiological connections. Acta Diabetol. 2021;58(8):997–1007. 10.1007/s00592-020-01668-5. 33743080 10.1007/s00592-020-01668-5 PMC8272710

[14] Czogała W, Strojny W, Schab M, Grabowska A, Miklusiak K, Kowalczyk W, Łazarczyk A, Tomasik P, Skoczeń S. FTO and PLAG1 genes expression and FTO methylation predict changes in Circulating levels of adipokines and Gastrointestinal peptides in children. Nutrients. 2021;13(10). 10.3390/nu13103585. 10.3390/nu13103585 PMC8538237 34684585

[15] Mounien L, Bizet P, Boutelet I, Vaudry H, Jégou S. Expression of melanocortin MC3 and MC4 receptor mRNAs by neuropeptide Y neurons in the rat arcuate nucleus. Neuroendocrinology. 2005;82(3–4):164–70. 10.1159/000091737. 16508337 10.1159/000091737

[16] Tao YX. The melanocortin-4 receptor: physiology, pharmacology, and pathophysiology. Endocr Rev. 2010;31(4):506–43. 10.1210/er.2009-0037. 20190196 10.1210/er.2009-0037 PMC3365848

[17] Qu H, Li J, Chen W, Li Y, Jiang Q, Jiang H, Huo J, Zhao Z, Liu B, Zhang Q. Differential expression of the melanocortin-4 receptor in male and female C57BL/6J mice. Mol Biol Rep. 2014;41(5):3245–56. 10.1007/s11033-014-3187-5. 24488261 10.1007/s11033-014-3187-5

[18] Mountjoy KG, Wild JM. Melanocortin-4 receptor mRNA expression in the developing autonomic and central nervous systems. Brain Res Dev Brain Res. 1998;107(2):309–14. 10.1016/s0165-3806(98)00015-7. 9593962 10.1016/s0165-3806(98)00015-7

[19] Wang Y, Bernard A, Comblain F, Yue X, Paillart C, Zhang S, Reiter JF, Vaisse C. Melanocortin 4 receptor signals at the neuronal primary cilium to control food intake and body weight. J Clin Invest. 2021;131(9). 10.1172/jci142064. 10.1172/JCI142064 PMC8087202 33938449

[20] Widiker S, Karst S, Wagener A, Brockmann GA. High-fat diet leads to a decreased methylation of the Mc4r gene in the obese BFMI and the lean B6 mouse lines. J Appl Genet. 2010;51(2):193–7. 10.1007/bf03195727. 20453306 10.1007/BF03195727

[21] Mankowska M, Nowacka-Woszuk J, Graczyk A, Ciazynska P, Stachowiak M, Switonski M. Polymorphism and methylation of the *MC4R* gene in obese and non-obese dogs. Mol Biol Rep. 2017;44(4):333–9. 10.1007/s11033-017-4114-3. 28755272 10.1007/s11033-017-4114-3 PMC5579139

[22] Aykut A, Özen S, Gökşen D, Ata A, Onay H, Atik T, et al. Melanocortin 4 receptor (*MC4R*) gene variants in children and adolescents having familial early-onset obesity: genetic and clinical characteristics. Eur J Pediatr. 2020;179(9):1445–52. 10.1007/s00431-020-03630-7. 32185475 10.1007/s00431-020-03630-7 PMC7223532

[23] Kwon EJ, You YA, Park B, Ha EH, Kim HS, Park H, et al. Association between the DNA methylations of POMC, *MC4R*, and HNF4A and metabolic profiles in the blood of children aged 7–9 years. BMC Pediatr. 2018;18(1):121. 10.1186/s12887-018-1104-0. 29598821 10.1186/s12887-018-1104-0 PMC5877386

[24] Chen J, Chen L, Sanseau P, Freudenberg JM, Rajpal DK. Significant obesity-associated gene expression changes occur in the stomach but not intestines in obese mice. Physiol Rep. 2016. 10.14814/phy2.12793. 27207783 10.14814/phy2.12793 PMC4886165

[25] Seelig E, Henning E, Keogh JM, Gillett D, Shin E, Buscombe J, van der Klaauw AA, Farooqi IS. Obesity due to melanocortin 4 receptor (MC4R) deficiency is associated with delayed gastric emptying. Clin Endocrinol. 2022;96(2):270–5. 10.1111/cen.14615. 10.1111/cen.14615 34694010

[26] Pucci A, Batterham RL. Endocrinology of the Gut and the Regulation of Body Weight and Metabolism. In: *Endotext.* edn. Edited by Feingold KR, Anawalt B, Blackman MR, Boyce A, Chrousos G, Corpas E, de Herder WW, Dhatariya K, Dungan K, Hofland J South Dartmouth (MA): MDText.com, Inc. Copyright © 2000–2023, MDText.com, Inc.; 2000.

[27] Hammad MM, Abu-Farha M, Hebbar P, Cherian P, Al Khairi I, Melhem M, Alkayal F, Alsmadi O, Thanaraj TA, Al-Mulla F, et al. MC4R variant rs17782313 associates with increased levels of DNAJC27, Ghrelin, and visfatin and correlates with obesity and hypertension in a Kuwaiti cohort. Front Endocrinol (Lausanne). 2020;11:437. 10.3389/fendo.2020.00437. 32733386 10.3389/fendo.2020.00437 PMC7358550

[28] Lamiquiz-Moneo I, Mateo-Gallego R, Bea AM, Dehesa-García B, Pérez-Calahorra S, Marco-Benedí V, Baila-Rueda L, Laclaustra M, Civeira F, Cenarro A. Genetic predictors of weight loss in overweight and obese subjects. Sci Rep. 2019;9(1):10770. 10.1038/s41598-019-47283-5. 31341224 10.1038/s41598-019-47283-5 PMC6656717

[29] German J, Cordioli M, Tozzo V, Urbut S, Arumäe K, Smit RAJ, et al. Association between plausible genetic factors and weight loss from GLP1-RA and bariatric surgery. Nat Med. 2025. 10.1038/s41591-025-03645-3. 40251273 10.1038/s41591-025-03645-3 PMC12283387

[30] Smemo S, Tena JJ, Kim KH, Gamazon ER, Sakabe NJ, Gómez-Marín C, Aneas I, Credidio FL, Sobreira DR, Wasserman NF, et al. Obesity-associated variants within FTO form long-range functional connections with IRX3. Nature. 2014;507(7492):371–5. 10.1038/nature13138. 24646999 10.1038/nature13138 PMC4113484

[31] Doaei S, gholamalizadeh M, Jarrahi AM, Badakhanian M, Najafi R. The IRX3 gene; the missing link between the Fto gene and obesity. Clin Nutr. 2018;37:S266–7. 10.1016/j.clnu.2018.06.1937.

[32] Ponce-Gonzalez JG, Martínez-Ávila Á, Velázquez-Díaz D, Perez-Bey A, Gómez-Gallego F, Marín-Galindo A, et al. Impact of the FTO gene variation on appetite and fat oxidation in young adults. Nutrients. 2023. 10.3390/nu15092037. 37432153 10.3390/nu15092037 PMC10181223

[33] Landgraf K, Scholz M, Kovacs P, Kiess W, Körner A. FTO obesity risk variants are linked to adipocyte IRX3 expression and BMI of children - relevance of FTO variants to defend body weight in lean children? PLoS One. 2016;11(8):e0161739. 10.1371/journal.pone.0161739. 27560134 10.1371/journal.pone.0161739 PMC4999231

[34] Yilmaz Z, Davis C, Loxton NJ, Kaplan AS, Levitan RD, Carter JC, Kennedy JL. Association between MC4R rs17782313 polymorphism and overeating behaviors. Int J Obes (Lond). 2015;39(1):114–20. 10.1038/ijo.2014.79. 24827639 10.1038/ijo.2014.79 PMC4232480

[35] Park S, Daily JW, Zhang X, Jin HS, Lee HJ, Lee YH. Interactions with the MC4R rs17782313 variant, mental stress and energy intake and the risk of obesity in genome epidemiology study. Nutr Metabolism. 2016;13:38. 10.1186/s12986-016-0096-8. 10.1186/s12986-016-0096-8 PMC4875620 27213003

[36] Acosta A, Camilleri M, Shin A, Carlson P, Burton D, O’Neill J, Eckert D, Zinsmeister AR. Association of melanocortin 4 receptor gene variation with satiation and gastric emptying in overweight and obese adults. Genes Nutr. 2014;9(2):384. 10.1007/s12263-014-0384-8. 24458996 10.1007/s12263-014-0384-8 PMC3968288

[37] WHO. Obesity and Overweight: Key Facts. World Health Organization. 2024, Available at: https://www.who.int/news-room/fact-sheets/detail/obesity-and-overweight. Accessed 25 Oct 2025.

[38] Eisenberg D, Shikora SA, Aarts E, Aminian A, Angrisani L, Cohen RV, de Luca M, Faria SL, Goodpaster KPS, Haddad A, et al. 2022 American society of metabolic and bariatric surgery (ASMBS) and international federation for the surgery of obesity and metabolic disorders (IFSO) indications for metabolic and bariatric surgery. Obes Surg. 2023;33(1):3–14. 10.1007/s11695-022-06332-1. 36336720 10.1007/s11695-022-06332-1 PMC9834364

[39] Livak KJ, Schmittgen TD. Analysis of relative gene expression data using real-time quantitative PCR and the 2(-Delta delta C(T)) method. Methods. 2001;25(4):402–8. 10.1006/meth.2001.1262. 11846609 10.1006/meth.2001.1262

[40] Gowayed MA, El Achy S, Kamel MA, El-Tahan RA. Polymyxin B prevents the development of adjuvant arthritis via modulation of TLR/Cox-2 signaling pathway. Life Sci. 2020;259:118250. 10.1016/j.lfs.2020.118250. 32791152 10.1016/j.lfs.2020.118250

[41] Mahmoud R, Kimonis V, Butler MG. Genetics of obesity in humans: A clinical review. Int J Mol Sci. 2022;23(19). 10.3390/ijms231911005. 10.3390/ijms231911005 PMC9569701 36232301

[42] Wardle J, Carnell S, Haworth CM, Plomin R. Evidence for a strong genetic influence on childhood adiposity despite the force of the obesogenic environment. Am J Clin Nutr. 2008;87(2):398–404. 10.1093/ajcn/87.2.398. 18258631 10.1093/ajcn/87.2.398

[43] Loos RJF, Yeo GSH. The genetics of obesity: from discovery to biology. Nat Rev Genet. 2022;23(2):120–33. 10.1038/s41576-021-00414-z. 34556834 10.1038/s41576-021-00414-z PMC8459824

[44] Loos RJF. Genetic causes of obesity: mapping a path forward. Trends Mol Med. 2025;31(4):319–25. 10.1016/j.molmed.2025.02.002. 40089418 10.1016/j.molmed.2025.02.002

[45] Sanghera DK, Bejar C, Sharma S, Gupta R, Blackett PR. Obesity genetics and cardiometabolic health: potential for risk prediction. Diabetes Obes Metab. 2019;21(5):1088–100. 10.1111/dom.13641. 30667137 10.1111/dom.13641 PMC6530772

[46] Singh RK, Kumar P, Mahalingam K. Molecular genetics of human obesity: a comprehensive review. CR Biol. 2017;340(2):87–108. 10.1016/j.crvi.2016.11.007. 10.1016/j.crvi.2016.11.007 28089486

[47] Ren X, Tang X, Huang T, Hu Z, Wang Y, Zhou Y. FTO plays a crucial role in Gastrointestinal cancer and May be a target for immunotherapy: an updated review. Front Oncol. 2023;13:1241357. 10.3389/fonc.2023.1241357. 37916161 10.3389/fonc.2023.1241357 PMC10616962

[48] Yin D, Li Y, Liao X, Tian D, Xu Y, Zhou C, Liu J, Li S, Zhou J, Nie Y, et al. FTO: a critical role in obesity and obesity-related diseases. Br J Nutr. 2023;130(10):1657–64. 10.1017/S0007114523000764. 36944362 10.1017/S0007114523000764

[49] Popović AM, Huđek Turković A, Žuna K, Bačun-Družina V, Rubelj I, Matovinović M. FTO gene polymorphisms at the crossroads of metabolic pathways of obesity and epigenetic influences. Food Technol Biotechnol. 2023;61(1):14–26. 10.17113/ftb.61.01.23.7594. 37200795 10.17113/ftb.61.01.23.7594 PMC10187569

[50] Matsuo T, Nakata Y, Hotta K, Tanaka K. The FTO genotype as a useful predictor of body weight maintenance: initial data from a 5-year follow-up study. Metabolism. 2014;63(7):912–7. 10.1016/j.metabol.2014.03.013. 24798613 10.1016/j.metabol.2014.03.013

[51] Lotta LA, Mokrosiński J, Mendes de Oliveira E, Li C, Sharp SJ, Luan J, Brouwers B, Ayinampudi V, Bowker N, Kerrison N, et al. Human Gain-of-Function MC4R variants show signaling bias and protect against obesity. Cell. 2019;177(3):597–e607599. 10.1016/j.cell.2019.03.044. 31002796 10.1016/j.cell.2019.03.044 PMC6476272

[52] Cheraghi S, Mobaderi T, Mottaghi A, Movahedi Motlagh F, Taghizadeh S, Eghbali M. Genetic variants in the MC4R gene and risk of obesity/overweight: a systematic review and meta-analysis. Diabetes Obes Metab. 2025;27(7):3901–20. 10.1111/dom.16425. 40302631 10.1111/dom.16425

[53] Imangaliyeva A, Sikhayeva N, Bolatov A, Utupov T, Romanova A, Akhmetollayev I, et al. Genetic insights into severe obesity: a case study of MC4R variant identification and clinical implications. Genes. 2025;16(5):508. 40428329 10.3390/genes16050508 PMC12111737

[54] Yilmaz Z, Davis C, Loxton NJ, Kaplan AS, Levitan RD, Carter JC, Kennedy JL. Association between MC4R rs17782313 polymorphism and overeating behaviors. Int J Obes. 2015;39(1):114–20. 10.1038/ijo.2014.79. 10.1038/ijo.2014.79 PMC4232480 24827639

[55] Perez-Luque E, Daza-Hernandez ES, Figueroa-Vega N, Cardona-Alvarado MI, Muñoz-Montes N, Martinez-Cordero C. Interaction effects of FTO and MC4R polymorphisms on total body weight loss, post-surgery weight, and post-body mass index after bariatric surgery. Genes. 2024. 10.3390/genes15040391. 38674326 10.3390/genes15040391 PMC11049276

[56] Gozen O, Aypar B, Ozturk Bintepe M, Tuzcu F, Balkan B, Koylu EO, Kanit L, Keser A. Chronic nicotine consumption and withdrawal regulate melanocortin receptor, CRF, and CRF receptor mRNA levels in the rat brain. Brain Sci. 2024;14(1):63. 38248278 10.3390/brainsci14010063 PMC10813117

[57] Rastogi A, Qiu R, Campoli R, Altayeh U, Arriaga S, Khan MJ, et al. The role of fat mass and obesity-associated (FTO) gene in non-small cell lung cancer tumorigenicity and EGFR tyrosine kinase inhibitor resistance. Biomedicines. 2025. 10.3390/biomedicines13071653. 40722726 10.3390/biomedicines13071653 PMC12292738

[58] Na ES. Epigenetic mechanisms of obesity: insights from Transgenic animal models. Life. 2025;15(4):653. 40283207 10.3390/life15040653 PMC12028693

[59] Tsai PC, Glastonbury CA, Eliot MN, Bollepalli S, Yet I, Castillo-Fernandez JE, Carnero-Montoro E, Hardiman T, Martin TC, Vickers A, et al. Smoking induces coordinated DNA methylation and gene expression changes in adipose tissue with consequences for metabolic health. Clin Epigenetics. 2018;10(1):126. 10.1186/s13148-018-0558-0. 30342560 10.1186/s13148-018-0558-0 PMC6196025

[60] Andrade S, Morais T, Sandovici I, Seabra AL, Constância M, Monteiro MP. Adipose tissue epigenetic profile in Obesity-Related Dysglycemia - A systematic review. Front Endocrinol (Lausanne). 2021;12:681649. 10.3389/fendo.2021.681649. 34290669 10.3389/fendo.2021.681649 PMC8288106

[61] Fansa S, Acosta A. The melanocortin-4 receptor pathway and the emergence of precision medicine in obesity management. Diabetes Obes Metab. 2024;26(2):46–63. 10.1111/dom.15555. 38504134 10.1111/dom.15555 PMC11893075

[62] Somala CS, Sathyapriya S, Bharathkumar N, Anand T, Mathangi DC, Saravanan KM. Therapeutic potential of FTO demethylase in metabolism and disease pathways. Protein J. 2025;44(1):21–34. 10.1007/s10930-025-10250-3. 39923206 10.1007/s10930-025-10250-3

