# Supplementary material for: In Vivo Gastric Expression of FTO and MC4R in Sleeve Gastrectomy Patients: Diagnostic Utility Without Predictive Value for Weight Loss
Source: Obes Surg. 2025 Dec 9;36(1):137–50. doi: 10.1007/s11695-025-08399-y (PMC12852160; doi:10.1007/s11695-025-08399-y)
Supplement: Supplementary file 1 — Supplementary Material 1 (DOCX 139 KB) [file 11695_2025_8399_MOESM1_ESM.docx]

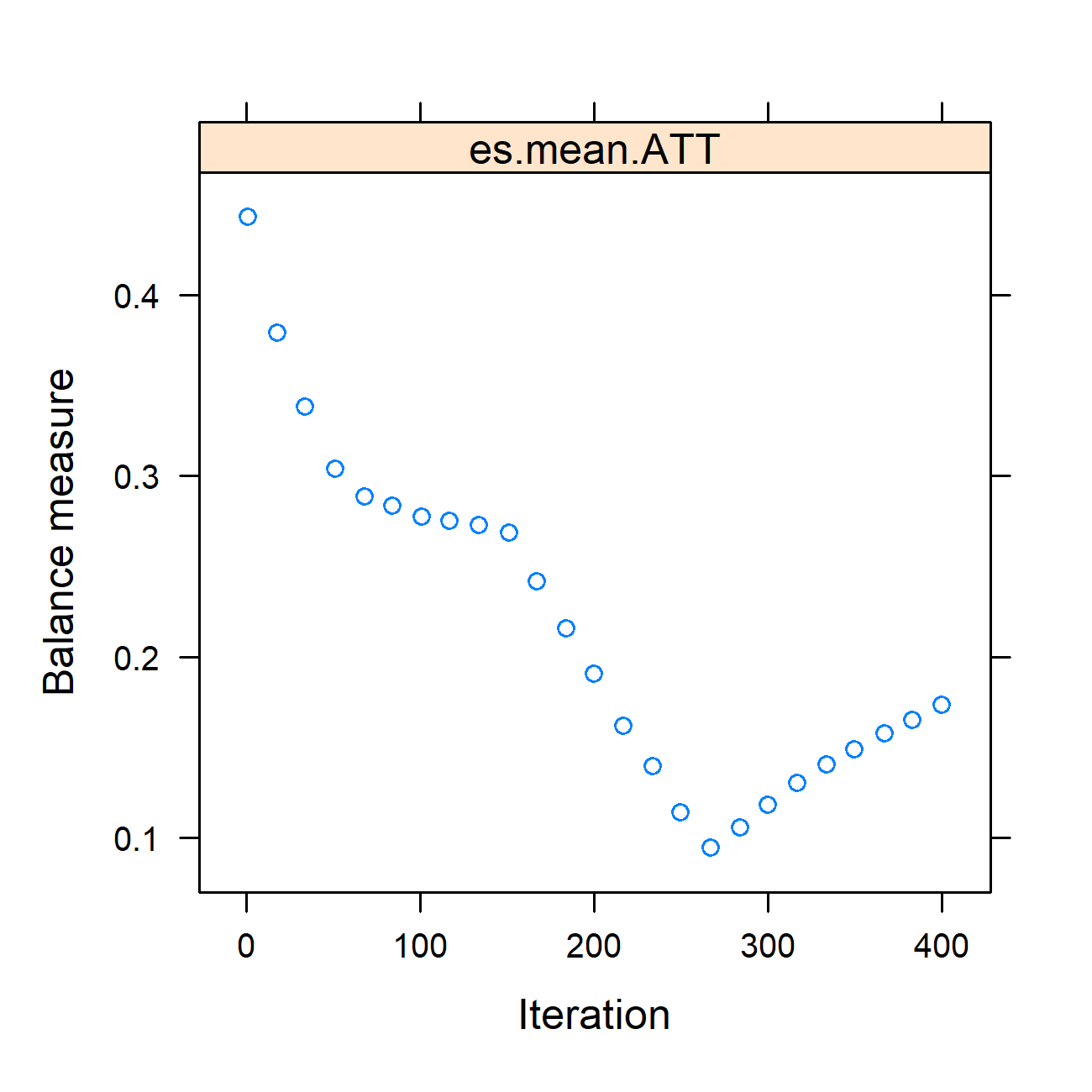


**Supplementary Figure S1**. Iterative balance improvement during the weighting process. The plot depicts the balance measure (absolute standardized mean difference, aSMD) across iterations during the estimation of inverse propensity score weights (IPSW). The balance measure initially decreases, reaching a minimum before slightly increasing in later iterations. The final weights were selected at the point of optimal balance, ensuring the best possible adjustment of covariates while maintaining sufficient sample size for analysis.


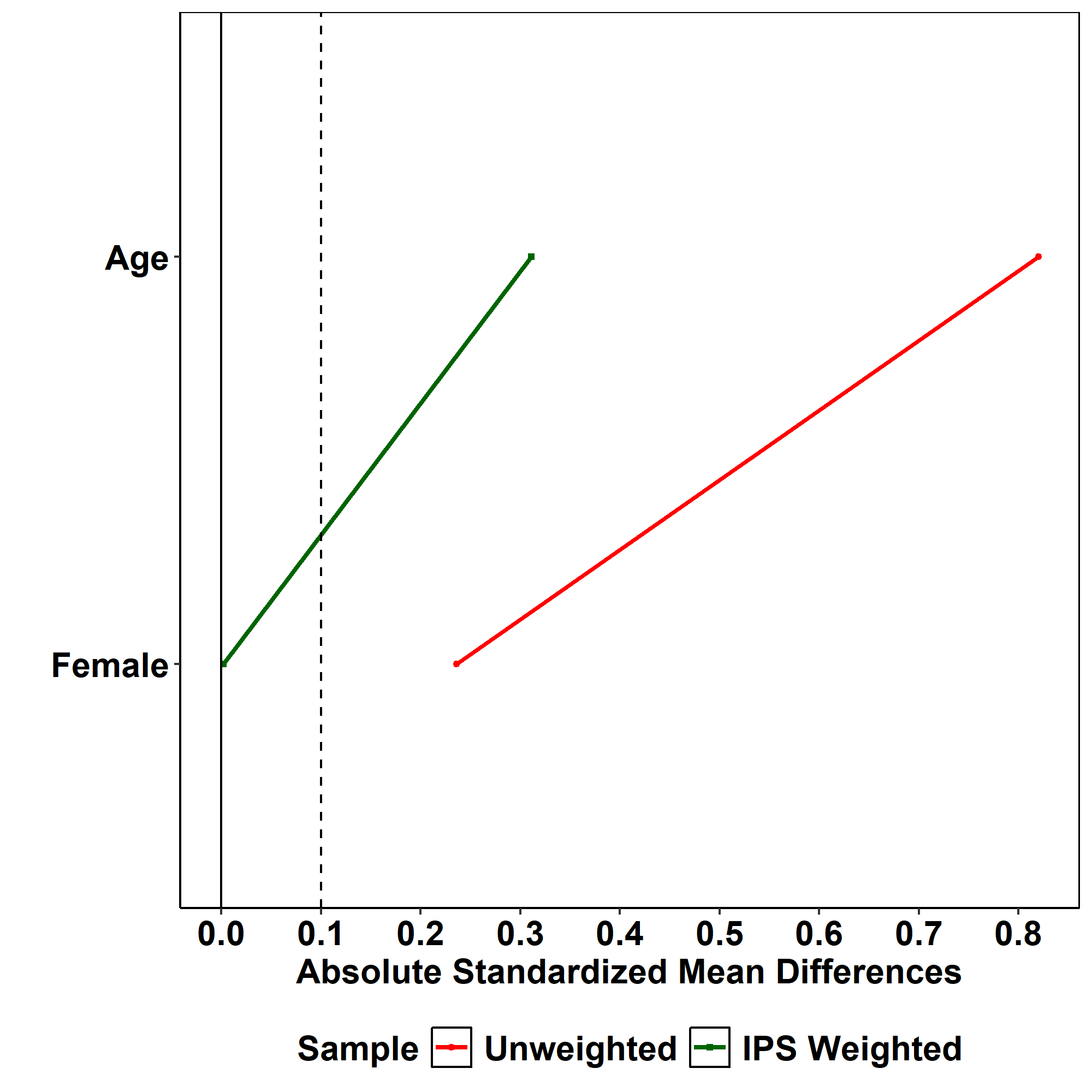


**Supplementary Figure S2. Standardized mean differences (SMD) before and after inverse propensity score weighting (IPSW).** The plot shows the absolute standardized mean differences (aSMD) for age and sex before (red line) and after (green line) IPSW adjustment. The dashed vertical line at 0.1 represents the threshold for optimal covariate balance. IPSW substantially reduced the imbalance in both variables, achieving an aSMD below 0.1 for sex, indicating improved comparability between cases and controls.


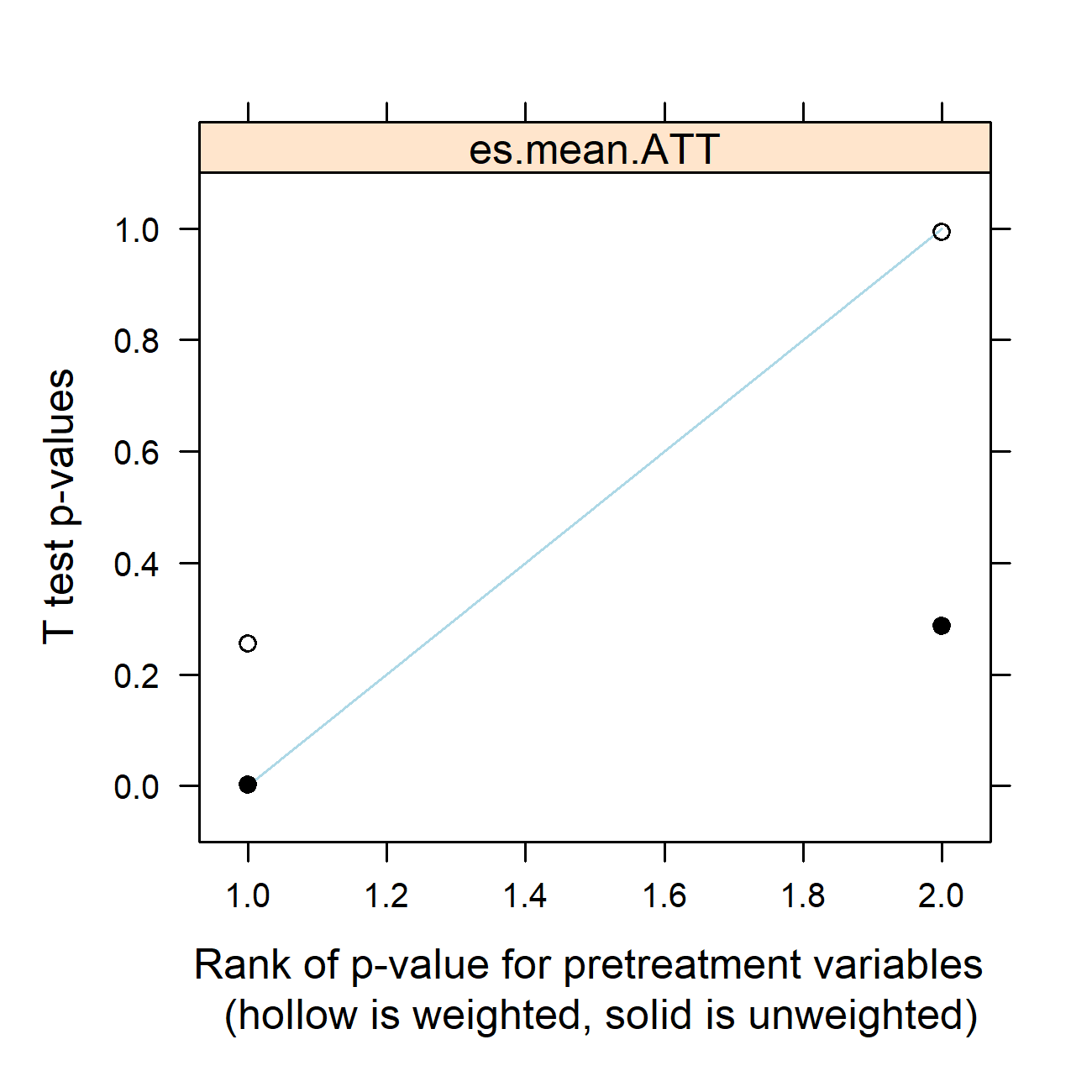


**Supplementary Figure S3. Comparison of p-values for covariate balance before and after inverse propensity score weighting (IPSW).** The solid points represent the p-values for the unweighted sample, while the hollow points correspond to the p-values after IPSW adjustment. The shift of points towards higher p-values after weighting demonstrates improved covariate balance between cases and controls, reducing the likelihood of significant baseline differences.


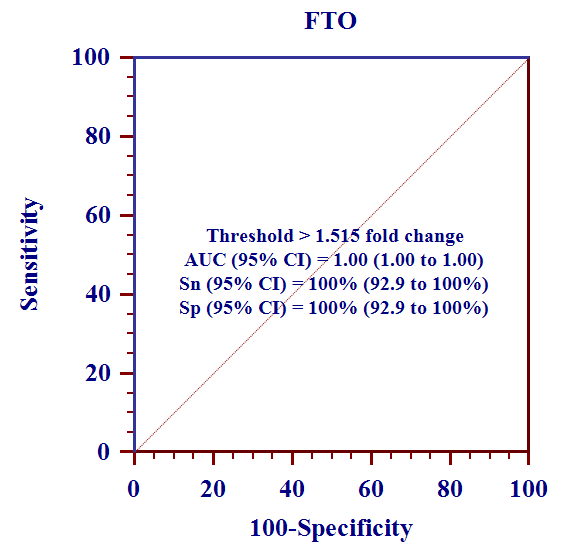


**Supplementary Figure S4. Receiver operating characteristic (ROC) curve for FTO gene expression (fold change) as a predictor of obesity.** The FTO threshold > 1.515 fold change was identified as the optimal cutoff, achieving an AUC of 1.00 (95% CI: 1.00 to 1.00), with both sensitivity and specificity reaching 100% (95% CI: 92.9 to 100%).


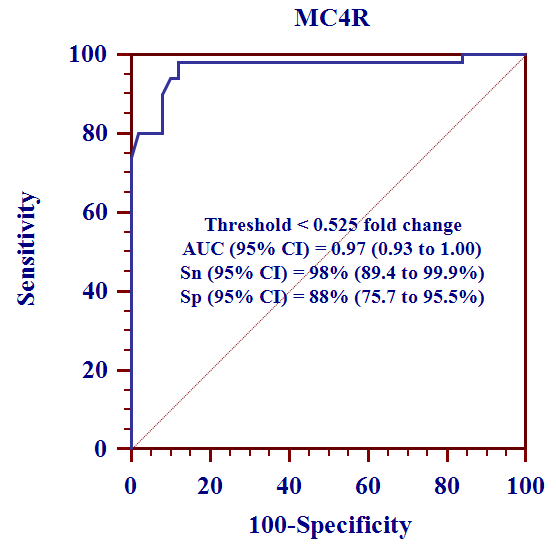


**Supplementary Figure S5. Receiver operating characteristic (ROC) curve for MC4R gene expression (fold change) as a predictor of obesity.** The MC4R threshold < 0.525 fold change was identified as the optimal cutoff, achieving an AUC of 0.97 (95% CI: 0.93 to 1.00), with a sensitivity of 98% (95% CI: 89.4 to 99.9%) and a specificity of 88% (95% CI: 75.7 to 95.5%).
